# Exploring the scope of 4sb and 12a lymph node dissection for cT2-4 lower third gastric cancer: Study protocol for a prospective cohort trial

**DOI:** 10.3389/fsurg.2022.956346

**Published:** 2022-07-14

**Authors:** Haiqiao Zhang, Zhi Zheng, Xiaoye Liu, Jun Cai, Jie Yin, Jun Zhang

**Affiliations:** Department of General Surgery, Beijing Friendship Hospital, Capital Medical University, Beijing, China

**Keywords:** 12a, lymph node dissection, lymph node metastasis, gastric cancer, 4sb

## Abstract

**Background:**

Currently, the extent of 4sb and 12a lymph node dissection is not clear and is based on the personal understanding of the surgeon. It may result in damage to the splenic artery and portal vein, leading to surgical complications. Therefore, this study aims to explore the scope of 4sb and 12a lymph node dissection in cT2-4 lower third gastric cancer.

**Methods:**

This is an ongoing prospective cohort trial. The total sample size required for the trial (March 2022 to February 2025) is approximately 524 patients. The participants are divided into the experimental (4sb first branch and 12a anterior lymphadenectomy) or control groups (traditional 4sb and 12a lymphadenectomy). Electronic data capture systems will be used to collect demographic, laboratory test, auxiliary examination, operation, postoperative condition, postoperative pathology, and follow-up data. The primary outcome is the 12a lymph node metastatic rate. Secondary outcomes include the pathology (consisting of the 4sb lymph node metastatic rate, the number of 4sb lymph nodes dissected, the number of 12a lymph nodes dissected and tumor pathological staging), a safety evaluation index (consisting of complications and mortality ≤30 days after surgery), an efficacy evaluation (consisting of operation data and postoperative recovery status), and follow-up data (consisting of 3-year or 5-year disease-free survival and overall survival).

**Discussion:**

By exploring the scope of 4sb and 12a lymph node dissection on the premise of ensuring radical cure of the tumor, the operation is simplified, the operation time is shortened, the damage of important blood vessels is reduced, the intraoperative and postoperative complications are reduced, and the patient recovers as soon as possible. Our study is a prospective exploration of the pathology, safety, efficacy, and prognosis of the new and traditional methods of 4sb and 12a lymph node dissection.

**Trial registration:**

Chinese Clinical Trial Registry, ChiCTR2200057698 (registration date: March 15, 2022).

## Introduction

Over the last few years, gastric cancer has ranked fifth in morbidity and fourth in mortality among all cancers globally (1). In 2020, the number of new cases and deaths from gastric cancer in China were 478,000 and 375,000 respectively, accounting for 43.9% and 48.6% of the global new cases and deaths from gastric cancer (2). The prognosis of gastric cancer is poor and the 5-year overall survival (OS) rate is only 35.9% in China (3). Lymph node metastasis (LNM) is the main metastasis of gastric cancer. Therefore, strict implementation of intraoperative lymphadenectomy is essential. According to the European Society of Medical Oncology (ESMO), National Cancer Comprehensive Network (NCCN), and Japanese Gastric Cancer Association (JGCA), D2 lymphadenectomy is considered as the standard treatment for gastric cancer. However, the extent of 4sb and 12a lymph node dissection (LND) is undefinable according to these guidelines (4–6), it tends to be based more on the surgeon's personal understanding and experience.

Adequate lymphadenectomy contributes to the radical cure of tumors and improves the prognosis. Concurrently, it improves the accuracy of postoperative pathological results and guides patients' postoperative treatment. However, A study has shown that excessive lymphadenectomy is associated with higher postoperative mortality (7). Therefore, lymphadenectomy should be controlled within an appropriate range. According to the guidelines (4–6), the scope of 4sb LND is along the left gastroepiploic artery (LGA), including the root, and the scope of 12a LND is along the proper hepatic artery (PHA), and needs to expose the anterior wall of the portal vein.

The advantage of the traditional lymphadenectomy is that it thoroughly cleans the regional lymph nodes (LNs). However, there are important blood vessels around the 4sb and 12a LNs, such as the splenic artery and portal vein. The risk of vascular damage is higher, the operation is difficult, and the operation time is long. Patients with advanced lower third gastric cancer have a low rate of 4sb and 12a LNM, and the prognosis is poor. Therefore, we designed a new cleaning range. We divided the 4sb LNs into two parts, namely those at the root of the LGA and the peripheral LNs of the first branch. 12a LNs were divided into two parts, namely the anterior and posterior peripheral LNs of the PHA. According to the patient's condition, we will take the 4sb LND area as the first branch LNs of the LGA, saving those at the root, and take the 12a LNs in the anterior area of the PHA, saving those in the posterior area, in some patients. The purpose is to simplify the operation, shorten the operation time, reduce the surgical complications, and speed up the recovery of the patient while obtaining the same radical cure effect. However, the efficacy and safety of this new technique is still unclear.

Since the scope of 4sb and 12a LND is not uniform nationally and internationally and there are few studies on the effectiveness and safety of different anatomical scopes, clinical study on the scope of 4sb and 12a LND is necessary. Therefore, we designed this experiment to compare the pathology, safety, efficacy, and prognosis of the new and traditional methods of 4sb and 12a lymphadenectomy to provide a new, alternative lymphadenectomy for patients with gastric cancer.

## Material and methods

### Study design

**Figure 1:**
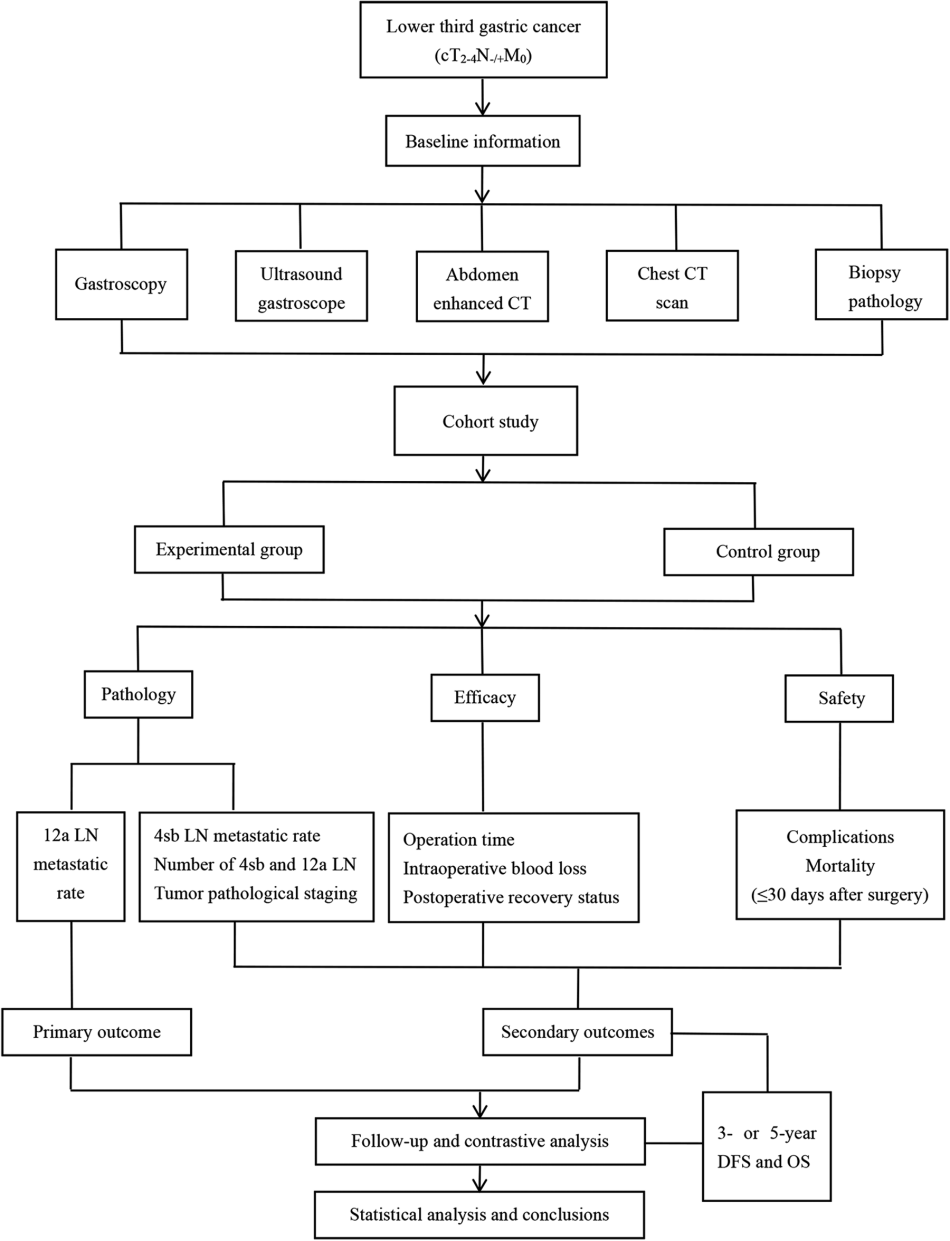
Research process and flow chart. LN, lymph node.

This is a single-center prospective cohort study. The study period is from March 2022 to February 2025. 524 patients will participate in the study. The participants are divided into the experimental (4sb first branch and 12a anterior lymphadenectomy) or control groups (traditional 4sb and 12a lymphadenectomy). After providing informed consent, the participants will be assigned for surgical treatment by the surgeon to either the experimental or control group at a 1:1 ratio. If the patients in the experimental group are found to have positive 4sb and 12a LNs during preoperative imaging examination or during the operation, they will be included in the control group for traditional lymphadenectomy. Figure 1 is a schematic diagram of the study process.

### Inclusion and exclusion criteria

Participants need to meet the following inclusion criteria: (I) gastroscopy biopsy pathologically diagnosed as gastric cancer, with clinical stage cT_2−4_N_−/+_M_0_ by abdominal enhanced computed tomography (CT) and ultrasound endoscopy in selected cases, and tumor location in the lower third of the stomach; (II) completion of laparoscopic distal gastric surgery with D2 lymphadenectomy, with or without conversion to laparotomy; (III) age 18–75 years, with no sex restrictions; (IV) cardiopulmonary function that can tolerate radical gastric cancer surgery; (V) ability to conform to the study protocol and sign the informed consent form; (VI) complete case information.

Patients that meet any of the following criteria are excluded from this study: (I) distant metastases found before or during the operation, and radical gastrectomy is not possible; (II) inability to tolerate anesthesia or surgery or refusal of surgery; (III) cerebrovascular injury or severe heart disease that have occurred within the past six months; (IV) a history of epilepsy, central nervous system disease or mental illness that inhibits cooperation with the study; (V) other diseases that seriously affect survival time; (VI) organ transplantation requiring immunosuppressive therapy; (VII) pregnancy or lactation; and (VIII) enrollment in other clinical study.

### Participating surgeons

The surgeon's proficiency and experience in radical gastric cancer surgery with D2 lymphadenectomy have been found to affect the number of LNs dissected, postoperative recovery time, and postoperative complications (8). A meta-analysis has shown that if surgeons can perform 40 operations alone, they will be able to independently manage any issues encountered during the operation and ensure the operational standards and safety (8). The study medical team will be composed of two experienced surgeons and several experienced nurses, who will be responsible for performing operative and postoperative medical therapy. The two surgeons each completed at least 60 cases of laparoscopic radical gastric distal surgery with D2 lymphadenectomy.

### Primary outcome

The primary outcome is the 12a lymph node metastatic rate. The 12a lymph node metastatic rate is equal to the number of patients with 12a LNM divided by the total number of patients enrolled.

### Secondary outcomes

The secondary outcome has four parts: pathology data, efficacy index, safety index, and follow-up data. The pathology data comprises the 4sb lymph node metastatic rate, the number of 4sb LNs dissected, the number of 12a LNs dissected, and tumor pathological staging. The efficacy evaluation index comprises operation time, intraoperative blood loss, and postoperative recovery status (time to get out of bed, time to pass gas, time to liquid food intake, time to semi-liquid food intake, and postoperative hospital stay). The safety evaluation index comprises complications and mortality ≤30 days after surgery. Intraoperative complications include injuries of the hepatic artery, common bile duct, portal vein, spleen and pancreas. Postoperative complications include biliary fistula, delayed hepatic artery and portal vein hemorrhage, pancreatic fistula, and other pancreatic injury-related complications. Follow-up data comprises 3-year or 5-year disease-free survival and OS.

### Interventions

First, all the patients will undergo laparoscopic surgery. Patients require gastroscopy biopsy pathology results to confirm the diagnosis, and endoscopic ultrasound and abdominal enhanced CT to evaluate the clinical stage and tumor location before surgery. To assess the general state of the patient and the risk of surgery, patients also need laboratory examinations including routine blood tests, blood biochemical tests, coagulation function tests, lung function tests, echocardiogram, and chest CT.

**Figure 2:**
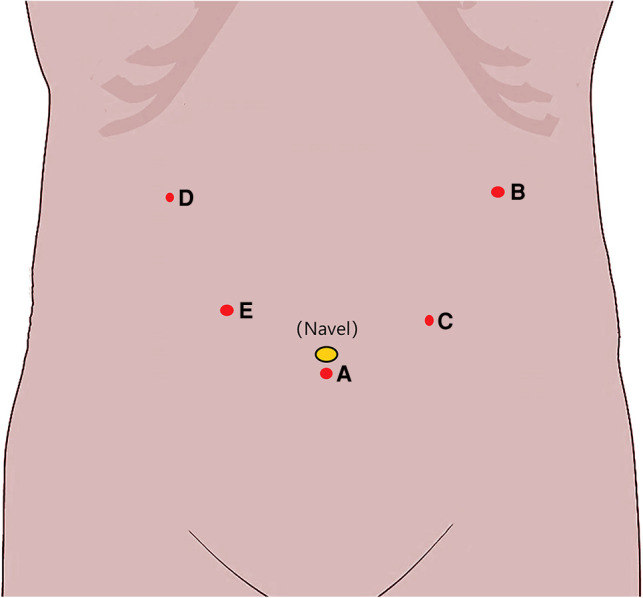
Puncture port placement.

All patients will undergo supine and split leg surgery. We will use the following methods for puncture port placement: (a) Insert a 12 mm trocar through a 1 cm transverse incision under the navel to establish a pneumoperitoneum and maintain the pressure at 12–15 mmHg. This opening will also be used for observation. (b) Insert a 12 mm trocar 2 cm below the costal margin of the left anterior axillary line as the surgeon's main operation opening. (c) Insert a 5 mm trocar 1 cm above the navel of the left mid-clavicular line as an auxiliary operating opening for the surgeon. (d) Insert a 5 mm trocar 2 cm below the costal margin of the right anterior axillary line as a secondary operation opening for the assistant. (e) Insert a 12 mm trocar 1 cm below the midpoint of the connection between opening A and opening D as the assistant's main operation opening. The location of the puncture opening is shown in Figure 2.

### Experimental group

We take the 4sb LNs at the first branch of the LGA and take the 12a LNs in the anterior area of the PHA. LNs in the two areas will be pathologically tested separately. The two groups of LNs will be separated and removed by the surgeon during the operation, rather than trimmed from the whole *ex vivo* pathological specimen.

**Figure 3:**
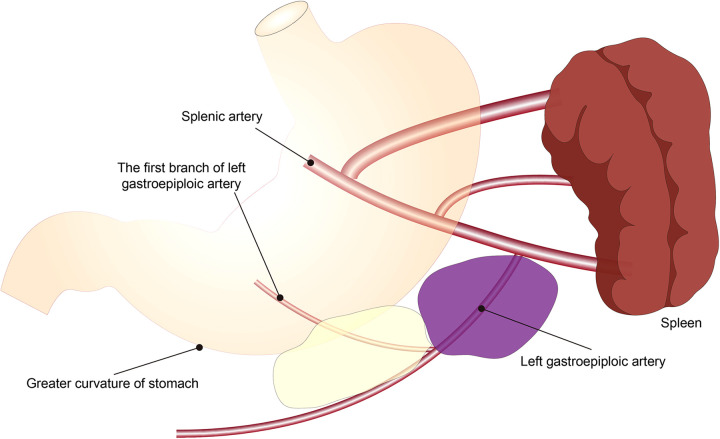
Schematic diagram of the scope of 4sb lymph node dissection. The yellow area is the experimental group, and the yellow plus purple area is the control group.

The scope of 4sb LND is as follows: we choose to cut off the LGA at the beginning of the first branch of the LGA, and then sweep along the greater curvature to the LGA and the right gastroepiploic artery interchange (Figure 3).

**Figure 4:**
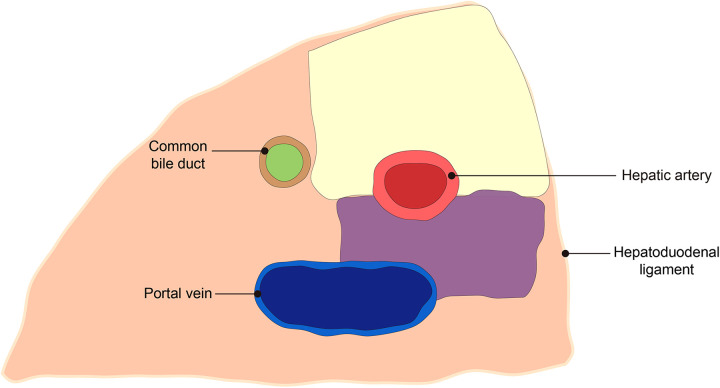
Schematic diagram of the scope of 12a lymph node dissection. The yellow area is the experimental group, and the yellow plus purple area is the control group.

The scope of 12a LND is as follows: (I) Upper border: the confluence of the right and left hepatic artery. (II) Lower border: the upper border of the pancreas at the origin of the PHA. (III) Right border: the left side of the bile duct. (IV) Left border: the left border of ligamentum hepatoduodenal. (V) Anterior border: the anterior hepatoduodenal ligament. (VI) Posterior border: the anterior side of the PHA (Figure 4).

### Control group

We take the 4sb LNs at the first branch and the root of the LGA and take 12a LNs in the anterior and posterior areas of the PHA. LNs in the two areas will be pathologically tested separately. The two groups of LNs will be separated and removed by the surgeon during the operation, rather than trimmed from the whole *ex vivo* pathological specimen.

The scope of 4sb LND is as follows: we choose to cut off the LGA at the root of the LGA originating from the splenic artery, and then sweep along the greater curvature to the LGA and the right gastroepiploic artery interchange (Figure 3).

The scope of 12a LND is as follows: posterior border is the anterior side of the portal vein. The remaining borders are the same as the experimental group (Figure 4).

### Perioperative treatment

Patients will receive symptom-based treatment after surgery, including electrocardiogram monitoring, intravenous fluid replacement, acid suppression, and pain relief. Routine blood tests, biochemical tests, measurement of drainage fluid amylase, and drainage fluid characteristics will be regularly monitored to observe whether bleeding, biliary fistula, and pancreatic injury-related postoperative complications occur. The discharge standards are as follows: gastrointestinal function is restored, the patient can have a semi-liquid diet, and the abdominal drainage tubes are removed.

### Data collection

We will use numbers instead of the name and sex of the enrolled patient to protect the privacy of the patient. Electronic data capture (EDC) system will be used to collect clinical data. The following data will be collected: (I) demographic, including sex, age, body mass index and concomitant disease; (II) perioperative laboratory test results, including those of routine blood and biochemical tests; (III) auxiliary examination results, including those of gastroscopy biopsy, endoscopic ultrasound and abdominal enhanced CT; (IV) surgical data, including operation time, blood loss, surgical method, extent of lymphadenectomy and intraoperative complications; (V) postoperative recovery data including time to get out of bed, time to pass gas, time to liquid food intake, time to semi-liquid food intake, postoperative hospital stay; and (VI) postoperative pathology, including pathological stage, the number of 4sb and 12a LNs dissected and metastasis rate (Table 1).

**Table 1:** Checklist for clinical data collection and follow-up plan of enrolled patients.

	Baseline		Post-operation					Follow-up							
	Before operation	Operation	POD1	POD3	POD5	POD7	POD30	3 m	6 m	12 m	18 m	24 m	36 m	48 m	60 m
Inclusion/Exclusion criteria	×														
Informed consent	×														
Demographic information	×														
Laboratory tests	×	×	×	×	×	×		×	×	×	×	×	×	×	×
Operation information		×													
Postoperative recovery status			×	×	×	×	×								
Physical examination	×							×	×	×	×	×	×	×	×
Chest CT	×									×		×	×	×	×
Abdomen enhanced CT	×							×	×	×	×	×	×	×	×
Gastroscopy	×									×		×	×	×	×
Ultrasound gastroscope	×														
Pathology	×					×									
Complications							×								
Mortality							×	×	×	×	×	×	×	×	×
Recurrence								×	×	×	×	×	×	×	×

*“×”, the need to collect the clinical data.*

*POD, postoperative day.*

### Follow-up

We will arrange for a full-time nurse to be responsible for enrolled patients postoperative follow-up in the hospital, and the outpatient clinic after discharge. The follow-up time points are 3, 6, 12, 18, 24, 36, 48, and 60 months. During the follow-up period, the patients need to receive physical and laboratory examinations, chest CT, abdominal enhanced CT, and gastroscopy. Laboratory examinations will include routine blood and biochemical tests (Table 1).

### Adverse events

A serious adverse event (SAE) refers to any adverse medical event, whether related to surgery or not. All SAEs will be recorded and reported to the ethics committee of Beijing Friendship Hospital within 24 h for enrolled patients. During the research period, the data monitoring committee will follow the standard procedures of clinical trials and supervise safety data in an unblinded manner.

Patients that have postoperative complications will receive the best therapy. Meanwhile, the enrollment will be suspended if the incidence of SAE exceeds 5% of enrolled patients. The Efficacy and Safety Evaluation Committee (ESEC) will evaluate whether to continue this study.

### Monitoring and quality assurance

The 4sb and 12a LNs removed during the operation fixed with 10% neutral formalin and pathological examinations performed. Standardized procedures for LNs pathology are: (I) The LNs were initially retrieved by the surgeon and completely immersed in a 10% neutral formalin specimen bag containing 6–8 times the specimen volume within half an hour of being isolated. The sample will then be sent to the pathology department for further lymph node retrieval by a pathologist. Soaking time: 12–48 h. (II) Paraffin section production process: (A) Taking materials: first, remove the LNs soaked in 10% neutral formalin from the specimen bag and then remove the fat tissue around the nodes. The larger LNs can be cut into slices every 0.5 cm in parallel. The smaller LNs can be opened along the axis. (B) Fixation: 10% neutral formalin fixation is used for routine paraffin sections. (C) Dehydration: From low-concentration alcohol to high-concentration alcohol, step by step replacing the alcohol in time to ensure the concentration, so that the water in the tissue is completely removed. (D) Soaking wax: use 58%–60% melting point paraffin for 2 h, replace paraffin once every hour, to embed wax use 60%–62% paraffin. (E) Slice: The slice thickness is generally 3 µm. Bake slices in a 60°C incubator for 40 min. (III) Immunochemistry: place the sections in water, keep them at room temperature with 3% neutral formalin for a certain period, and wash the sections with distilled water and clean water. Then place the slices in a pressure cooker containing 1.1 L citric acid with pH 6.0, heat for 2 min and cool naturally, then take out and rinse twice with phosphate buffer saline, 2 min each time, add secondary antibody until the diaminobenzidine staining solution develops the color and compare the negative and positive of the antibody. (IV) Reading: finally, observe under a microscope to determine whether there are abnormal cells in the LNs to determine whether there is LNM. Lymph node micrometastasis will be defined as node-positive.

A Data and Safety Monitoring Committee (DSMC) was established, and is composed of senior professors, data administrators, ethics experts and statistician. We will clearly assign responsibilities to committee members and they will cooperate with each other. The DSMC can access the research data and other records unrestrictedly for quality assurance activities. Meanwhile, emergency review and assessment of safety-related issues can also be conducted. The DSMC is independent of research funders and has no competing interests.

### Sample size

The sample size was calculated using PASS 11.0 software. The 12a lymph node metastatic rate was used as the main result in the sample size calculation. This study assumes that the 12a lymph node metastatic rate in the experimental group is not inferior to the control group. According to published literature (9), the 12a lymph node metastatic rate of the control group is estimated at 9.1%. Combined with clinical practice, the non-inferiority threshold is set to −7.4%. The size of the two groups of patients was determined with a 1:1 design, setting a significance level of *α* = 0.025 (unilateral), power at 80%, and loss to follow-up at 10%. We expect 524 patients to be registered, with 262 patients in each group.

### Statistical analysis

The normally distributed data will be expressed as mean ± standard deviation, and independent sample t-test will be used for groups differences. The non-normally distributed data will be expressed by a median, and the Mann–Whitney U test will be used for groups differences. The count data will be described as frequency and percentage and Fisher's exact test will be used for groups differences. The survival curve will be analyzed with the use of Kaplan–Meier, and the log-rank test will be used to evaluate groups differences. The Cox proportional hazards model will be used to evaluate the hazard ratio. *P* < 0.05 is set as the significance level.

### Interim analyses

When the number of enrolled patients reaches 262, we will perform statistical analysis on the primary and secondary outcomes. The independent statistical team will be responsible for the interim analysis and report the results to the DSMC. The DSMC will discuss the results and report to the ESEC. If the effectiveness and safety of the experimental group are significantly lower than that of the control group, the study will be suspended.

### Dissemination plans

We plan to publish the results of short-term efficacy and safety studies in 2025, and the results of the long-term follow-up studies in 2030.

### Trial status

The study was registered at the Chinese Clinical Trial Registry (http://www.chictr.org.cn), with number ChiCTR2200057698 on March 15, 2022. Currently, our research team has established the EDC systems to collect clinical data.

## Discussion

LNM is an important factor affecting the 5-year OS rate of gastric cancer patients. Therefore, the prognostic value of D2 lymphadenectomy in gastric cancer needs to be further improved (10). Saito (11) found that in 239 cases of lower-third gastric cancer undergoing D2 lymphadenectomy, the lymph node metastatic rate in the 4sb group was 2.2%, which was related to LNs invasion and advanced gastric cancer. Shu (12) found that the metastasis rate of 12a LNs was 2.67% (11/413), which was related to tumor location, depth of tumor invasion and LNs stage. For patients with advanced gastric cancer (stages III/IV) located in the lower third of the stomach, the metastasis rate of 12a LNs increased to 10.7% (11/103). The recurrence-free survival rate and OS rate of patients with 12a LNM were significantly worse. Other studies have shown that the metastasis rate of 12a LNs is 1.7%–20% and it may be one of the most important factors affecting prognosis (7, 9, 10, 13–15). Therefore, accurate lymphadenectomy plays a positive role in the prognosis. However, the scope of 4sb and 12a LND in D2 lymphadenectomy is not clear. Lin (16) found that the unqualified rate of lymphadenectomy in 948 cases of laparoscopic total gastrectomy was 51.9%, and it gradually decreased with accumulated surgical experience. It is believed that the main reason for the unqualified LNs failure is the failure to dissect LNs at specific locations during the operation. Among them, the failure rate of the 12a LNs was higher (33.44%), and the No.4 LNs were lower (9.39%). This shows that a standardized and clear range of lymphadenectomy is significantly helpful for clinicians treating gastric cancer and undertaking complete D2 lymphadenectomy. However, there are few studies on the cleaning range of 4sb and 12a.

Huang (17) pointed out that the posterior boundary of 12a LND was defined as the anterior edge of the portal vein, so only the LNs in front of the portal vein were cleaned during the operation. Huang (14) published a new 12a LND sequence, suggesting that the portal vein should be exposed first and the LNs before the portal vein should be cleaned. However, we considered that the 12a lymph node metastatic rate is lower for advanced lower third gastric cancer, and LNM around the portal vein is even more rare. Therefore, we suggested that only the LNs area in the front of the PHA should be cleaned, retaining the LNs area around the portal vein. The same is true for the 4sb LNs. We suggested cleaning the LNs around the first branch of the LGA and retaining the LNs around the root of the LGA. Through such a cleaning method, under the premise of ensuring the radical cure of the tumor, the risk of damage to blood vessels and organs, and complications during surgery, are reduced.

Although ESMO, NCCN and JGCA regularly update the guidelines for treating gastric cancer, they are all updated in terms of indications of the extent of LND, and there is no further detailed update of the definition of LND (4–6). In addition, although there have been some studies on 4sb and 12a LNs, the majority are related to LNM and risk factors or survival rates. Therefore, we are currently conducting a clinical trial to establish a safer 4sb and 12a LND range, to reduce the complication rate of D2 lymphadenectomy while obtaining the same radical cure effect.

## Conclusion

The limitations of this study are that the quality of evidence is lower than that of a randomized controlled study and there may be selection bias. However, we will build iterations of future study protocols based on the findings. In addition, we will also recruit more patients to determine more effective 4sb and 12a LND protocols and to provide ideas and evidence for lymphadenectomy in collaboration with other research centers.

## Data Availability

The original contributions presented in the study are included in the article/Suplementary Material, further inquiries can be directed to the corresponding author/s.
